# Primary tumor inflammation in gross tumor volume as a prognostic factor for nasopharyngeal carcinoma patients

**DOI:** 10.18632/oncotarget.7699

**Published:** 2016-02-25

**Authors:** Hao Peng, Lei Chen, Ling-Long Tang, Yuan Zhang, Wen-Fei Li, Yan-Ping Mao, Fan Zhang, Rui Guo, Li-Zhi Liu, Li Tian, Ai-Hua Lin, Ying Sun, Jun Ma

**Affiliations:** ^1^ Department of Radiation Oncology, Sun Yat-Sen University Cancer Center, State Key Laboratory of Oncology in Southern China, Collaborative Innovation Center for Cancer Medicine, Guangzhou, People's Republic of China; ^2^ Imaging Diagnosis and Interventional Center, Sun Yat-Sen University Cancer Center, State Key Laboratory of Oncology in Southern China, Collaborative Innovation Center for Cancer Medicine, Guangzhou, People's Republic of China; ^3^ Department of Medical Statistics and Epidemiology, School of Public Health, Sun Yat-Sen University, Guangzhou, People's Republic of China

**Keywords:** nasopharyngeal carcinoma, primary tumor inflammation, gross tumor volume, prognosis, intensity-modulated radiation therapy

## Abstract

**Purpose:**

The objective of this study is to investigate the prognostic value of primary tumor inflammation (PTI) in nasopharyngeal carcinoma (NPC) in the era of intensity-modulated radiation therapy (IMRT).

**Results:**

PTI was observed in 376/1708 (22.0%) patients, and was present in the sphenoid sinus in 289/376 (76.9%), in the nasal cavity in 27 (7.2%), and in both places in 60 (15.9%). The estimated 4-year local relapse-free survival (LRFS), disease-free survival (DFS), overall survival (OS) and distant metastasis-free survival (DMFS) rates for PTI vs. non-PTI group were 89.2% vs. 96.1% (*P* < 0.001), 73.4% vs. 85.1% (*P* < 0.001), 85.0% vs. 92.1% (*P* < 0.001) and 83.6% vs. 91.4% (*P* < 0.001), respectively. After adjustment for these known prognostic factors, PTI was confirmed as an independent prognostic factor for LRFS (HR 2.152, 95% CI 1.318–3.516, *P* = 0.002), DFS (HR 1.581, 95% CI 1.204–2.077, *P* = 0.001) and DMFS (HR 1.682, 95% CI 1.177–2.402, *P* = 0.004).

**Conclusions:**

Primary tumor inflammation was identified as a strong prognostic factor for patients with NPC in the era of IMRT and should be considered when devising future treatment strategies aimed at improving survival in NPC patients.

**Materials and Methods:**

Data on 1708 patients with nonmetastatic, histologically-confirmed NPC treated with IMRT between November 2009 and February 2012 at Sun Yat-Sen University Cancer Center were retrospectively reviewed. Patient survival between PTI and non-PTI groups were compared.

## INTRODUCTION

Worldwide, there were an estimated 84,400 new cases of nasopharyngeal carcinoma (NPC) and 51,600 deaths in 2011 [1]. NPC is an uncommon cancer with a very unique geographical distribution, with an age-standardized incidence rate of 20–50 per 100,000 males in south China but only 0·5 per 100,000 in Caucasian populations in 2011 [1]. Due to anatomic constraints and high radiosensitivity, radiotherapy is the only curative treatment for NPC. Despite its known limitations, the TNM staging system remains the most important prognostic factor for NPC patients [2]. Many other factors had been reported to have prognostic value, including plasma Epstein-Barr virus (EBV) DNA [3–5], primary tumor volume [6, 7], pretreatment serum lactate dehydrogenase (LDH) levels [8] and apparent diffusion coefficient (ADC) [9].

Due to the special location and invasiveness of NPC [10], primary tumor inflammation (PTI) in gross tumor volume (GTV) is a commonly imaging feature in advanced T stage patients. Using magnetic resonance (MR) imaging, inflammation in GTV is seen as an area of high signal intensity on T2-weighted images, and as an area of low signal intensity on contrast material-enhanced T1-weighted images. The prognostic value of necrosis in cervical nodal, a subtype of inflammation in NPC patients, has been demonstrated [11], as has the prognostic role of necrosis in bladder cancer [12]. Therefore, it is reasonable to speculate that the inflammation in GTV may also have prognostic value in NPC.

As we know, the relationship between primary tumor inflammation in GTV and prognosis of NPC patients has not been studied. Hence, we conducted a large-scale retrospective study to evaluate the impact of PTI on the clinical features and survival outcomes of NPC patients based on MR imaging (MRI) results.

## RESULTS

### Patient characteristics

Of the 1708 patients, the male (*n* = 1273)-to-female (*n* = 435) ratio was 2.9:1, the median age was 45 years (rang, 14–78 years). Patient characteristics are listed in Table 1. Of the 376 (22.0%) patients with PTI, 168 (44.7%) and 204 (54.3%) were classified as T_3_ and T_4_, respectively (*P* < 0.001), while only 4 (1.0%) were in disease stage T_1–2_. PTI was observed in the sphenoid sinus alone in 289 (76.9%) patients, in the nasal cavity alone in 27 (7.2%), and in both locations in 60 (15.9%). PTI and non-PTI groups were similar in terms of drinking (*P* = 0.262). However, the PTI group had a higher percentage patients that were older (*P* = 0.009), male (*P* = 0.035), smokers (*P* = 0.002), classified as advanced T (*P* < 0.001) and N (*P* < 0.001), and undergoing chemotherapy (*P* < 0.001).

**Table 1:** Baseline characteristics of the 1708 patients included in this study

Characteristics	PTI group	Non-PTI group	Total	*P*^a^
	No. (%)	No. (%)		
Total	376	1332	1708	
Age (years)				0.009
< 50	236 (62.8)	931 (69.9)	1167	
≥ 50	140 (37.2)	401 (30.1)	541	
Sex				0.035
Male	296 (78.7)	977 (73.3)	1273	
Female	80 (21.3)	355 (26.7)	435	
WHO pathology				0.001
Type I	6 (1.6)	3 (0.2)	9	
Type II/III	370 (98.4)	1329 (99.8)	1699	
Smoking				0.002
Yes	163 (43.4)	460 (34.5)	623	
No	213 (56.6)	872 (65.5)	1085	
Drinking				0.262
Yes	53 (14.1)	159 (11.9)	212	
No	323 (85.9)	1173 (88.1)	1496	
T classification^b^				< 0.001
T1	2 (0.5)	307 (23.1)	309	
T2	2 (0.5)	258 (19.4)	260	
T3	168 (44.7)	648 (48.6)	816	
T4	204 (54.3)	119 (8.9)	323	
N classification^b^				< 0.001
N0	33 (8.8)	253 (19.0)	286	
N1	242 (64.4)	764 (57.3)	1006	
N2	76 (20.2)	190 (14.3)	266	
N3	25 (6.6)	125 (9.4)	150	
Overall stage^b^				< 0.001
I	0 (0)	92 (6.9)	92	
II	2 (0.5)	353 (26.5)	355	
III	158 (42.0)	657 (49.3)	815	
IVA–IVB	216 (57.5)	230 (17.3)	446	
Chemotherapy				< 0.001
Yes	366 (97.3)	1113 (83.6)	1479	
No	10 (2.7)	219 (16.4)	229	

Abbreviations: PTI = primary tumor inflammation; WHO = World Health Organization.

a *P* values were calculated using chi-square or Fisher exact test as indicated.

b According to the 7th edition of the AJCC/UICC staging system.

### Patient failure patterns

The median follow-up time for the entire cohort was 49.9 months (range, 1.3–76.4 months), and 263 (15.4%) patients were lost to follow-up. Patterns of treatment failure and cause of death are summarized in Table 2. Up to the final follow-up, 39/376 (10.4%) PTI patients and 52/1332 (3.9%) non-PTI patients experienced local failure (*P* < 0.001), 14/376 (3.7%) PTI patients and 55/1332 (4.1%) non-PTI patients experienced regional failure (*P* = 0.724), 62/376 (16.5%) PTI patients and 113/1332 (8.5%) non-PTI patients developed distant metastases (*P* < 0.001). Moreover, 59/376 (15.7%) PTI patients and 108/1332 (8.1%) non-PTI patients died, and the majority of deaths were attributed to NPC.

**Table 2:** Treatment failure patterns and cause of death

Failure patterns	PTI group	Non-PTI group	*P*^a^
	No. (%)	No. (%)	
Local only	29 (7.7)	27 (2.0)	< 0.001
Local + regional	2 (0.5)	11 (0.8)	0.911
Local + distant	6 (1.6)	9 (0.7)	0.172
Local + regional + distant	2 (0.5)	5 (0.4)	1.000
Regional only	4 (1.1)	32 (2.4)	0.206
Regional + distant	6 (1.6)	7 (0.5)	0.024
Distant only	48 (12.8)	91 (6.8)	< 0.001
Total locoregional	49 (13.0)	92 (6.9)	< 0.001
Total distant	62 (16.5)	113 (8.5)	< 0.001
Total	97	183	
Cause of death			0.406
Cancer	52 (88.1)	90 (83.3)	
Non-cancer	7 (11.9)	18 (16.7)	
Total	59	108	

Abbreviations: PTI = primary tumor inflammation.

a *P* values were calculated using chi-square or Fisher exact test as indicated.

### Univariate and multivariate analysis

**Figure 1:**
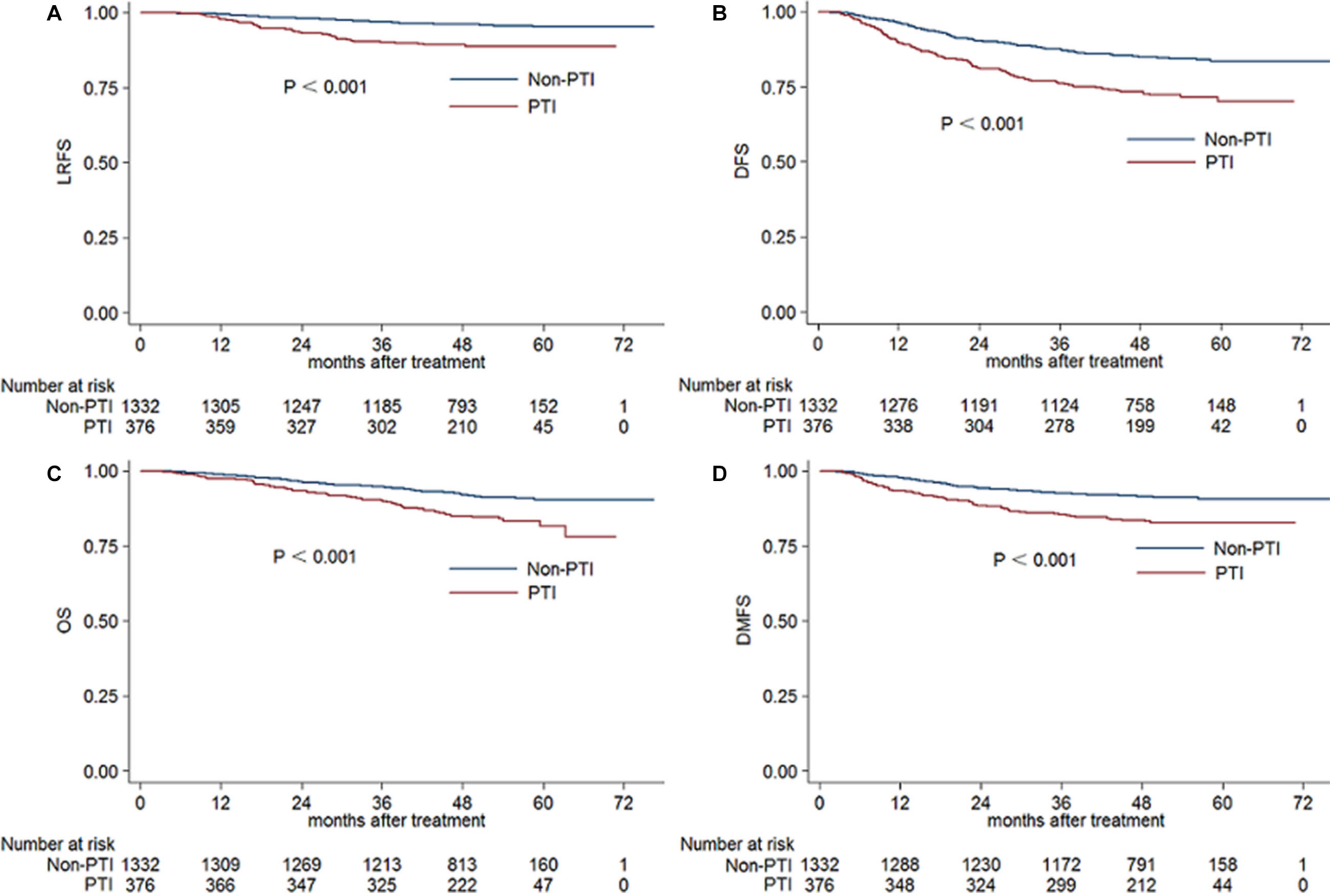
Kaplan-Meier LRFS (**A**), DFS (**B**), OS (**C**) and DMFS (**D**) curves for PTI and non-PTI patients. Abbreviations: LRFS = local relapse-free survival; DFS = disease-free survival; OS = overall survival; DMFS = distant metastasis-free survival; PTI = primary tumor inflammation.

The estimated 4-year local relapse-free survival (LRFS), disease-free survival (DFS), overall survival (OS) and distant metastasis-free survival (DMFS) rates for the whole cohort were 94.6%, 82.5%, 90.6% and 89.7%, respectively. For PTI group vs. non-PTI group, they were 89.2% vs. 96.1% (*P* < 0.001), 73.4% vs. 85.1% (*P* < 0.001), 85.0% vs. 92.1% (*P* < 0.001) and 83.6% vs. 91.4% (*P* < 0.001), respectively (Figure 1).

The results of univariate analysis revealed that there was prognostic value in age greater than 50 years, pathology type, PTI, T and N classification, and overall stage. PTI, T classification and overall stage were associated with 4-year LRFS, DFS, OS and DMFS (Table 3). After adjusting for various prognostic factors, the outcomes of multivariate analysis showed that PTI was an independent prognostic factor for LRFS (HR 2.152, 95% CI 1.318– 3.516, *P* = 0.002), DFS (HR 1.581, 95% CI 1.204–2.077, *P* = 0.001) and DMFS (HR 1.682, 95% CI 1.177–2.402, *P* = 0.004) (Table 4).

**Table 3:** Univariate analysis of variables associated with prognostic outcomes

Characteristics	Patients	4-year LRFS	*P*^a^	4-year DFS	*P*^a^	4-year OS	*P*^a^	4-year DMFS	*P*^a^
	No. (%)	(%)		(%)		(%)		(%)	
Age (years)			0.329		0.002		< 0.001		0.296
< 50	1167 (68.3)	94.9		84.4		92.6		90.2	
≥ 50	541 (31.7)	94.0		78.3		86.0		88.5	
Sex			0.105		0.876		0.143		0.276
Male	1273 (74.5)	95.1		82.4		89.9		89.2	
Female	435 (25.5)	93.2		82.6		92.5		91.2	
WHO pathology			< 0.001		0.011		0.008		0.353
Type I	9 (0.5)	66.7		55.6		66.7		100	
Type II/III	1699 (99.5)	94.8		82.6		90.7		89.6	
Primary tumor			< 0.001		< 0.001		< 0.001		< 0.001
PTI	376 (22.0)	89.2		73.4		85.0		83.6	
Non-PTI	1332 (78.0)	96.1		85.1		92.1		91.4	
Smoking			0.603		0.017		0.005		0.102
Yes	623 (36.5)	94.3		79.6		88.1		88.0	
No	1085 (63.5)	94.8		84.2		92.0		90.6	
Drinking			0.176		0.287		0.174		0.687
Yes	212 (12.4)	96.4		79.1		88.5		88.5	
No	1496 (87.6)	94.4		83.0		90.9		89.8	
T classification			< 0.001		< 0.001		< 0.001		< 0.001
T1	309 (18.1)	97.2		88.7		96.9		93.6	
T2	260 (15.2)	95.1		84.7		93.6		91.8	
T3	816 (47.8)	95.6		83.1		90.5		90.0	
T4	323 (18.9)	89.0		73.3		82.4		83.5	
N classification			0.552		< 0.001		< 0.001		< 0.001
N0	286 (16.7)	95.6		92.1		95.9		95.8	
N1	1006 (58.9)	94.5		84.2		92.8		92.0	
N2	266 (15.6)	93.3		75.5		85.3		83.7	
N3	150 (8.8)	96.0		64.3		74.2		72.2	
Overall stage			0.002		< 0.001		< 0.001		< 0.001
I	92 (5.4)	98.7		97.6		98.9		98.9	
II	355 (20.8)	95.7		88.6		94.6		94.6	
III	815 (47.7)	95.5		84.4		91.4		91.4	
IV	446 (26.1)	91.0		70.9		80.6		80.6	

Abbreviations: PTI = primary tumor inflammation; LRFS = local relapse-free survival; DFS = disease-free survival; OS = overall survival; DMFS = distant metastases-free survival; WHO = world health organization.

a *P* values were calculated using the log-rank test.

**Table 4:** Multivariate analysis of prognostic factors correlated with clinical outcomes

Endpoint	Variable	*P*^a^	HR	95% CI for HR
LRFS	PTI	0.002	2.152	1.318–3.516
	Pathology	0.002	0.163	0.051–0.552
DFS	Age	0.014	1.338	1.061–1.686
	PTI	0.001	1.581	1.204–2.077
	T classification	0.019	1.185	1.029–1.366
	N classification	< 0.001	1.656	1.455–1.884
OS	Age	< 0.001	1.778	1.309–2.414
	T classification	< 0.001	1.759	1.455–2.125
	N classification	< 0.001	1.943	1.638–2.304
DMFS	PTI	0.004	1.682	1.177–2.402
	N classification	< 0.001	2.013	1.705–2.377

Abbreviations: PTI = primary tumor inflammation; LRFS = local relapse-free survival; DFS = disease-free survival; OS = overall survival; DMFS = distant metastases-free survival; HR = hazard ratio; CI = confidence interval.

a Multivariate *P*-values were calculated using an adjusted Cox proportional-hazards model with backward elimination and the following parameters: age (≥ 50 y vs. < 50 y), gender (male or female), pathological type (type I or type II/III), PTI (yes or no), smoking (yes or no), drinking (yes or no), T classification, N classification, undergoing chemotherapy (yes or No).

### Subgroup analysis

Due to the unbalanced distribution of PTI in T classification, subgroup analysis according to T and N classification and overall stage was performed to further investigate the prognostic value of PTI (Table 5). The survival outcomes for PTI patients classified as T3 were substantially poorer than those T3 patients in the no-PTI group (*P* = 0.001 for LRFS, *P* = 0.005 for DFS, *P* = 0.041 for DMFS), and were similar to those of patients classified asT4 in the no-PTI group (90.6% vs. 90.9 for LRFS, 76.6% vs. 77.8% for DFS, 86% vs. 86.7% for DMFS). Additionally, for patients with N1 disease, the presence of PTI markedly affected prognosis (*P* < 0.05 for all rates). However, the presence of PTI did not substantially affect the outcomes of patients with T4 and N2-3 disease (*P* > 0.05 for all rates).

**Table 5:** Subgroup analysis of T, N and overall stage of PTI and non-PTI groups

Stage	4-year LRFS			4-year DFS			4-year OS			4-year DMFS		
	PTI (%)	Non-PTI (%)	*P*^a^	PTI (%)	Non-PTI (%)	*P*^a^	PTI (%)	Non-PTI (%)	*P*^a^	PTI (%)	Non-PTI (%)	*P*^a^
T												
T1	100	97.2	0.787	50	88.9	0.059	100	96.9	0.801	50	93.9	0.006
T2	100	95.1	0.740	100	84.6	0.558	100	93.6	0.716	100	91.7	0.678
T3	90.6	96.9	0.001	76.6	84.7	0.005	89	90.9	0.181	86	91	0.041
T4	87.9	90.9	0.331	70.7	77.8	0.138	81.5	84	0.954	81.7	86.7	0.192
N												
N0	90.9	96.2	0.153	81.8	93.5	0.022	89.8	96.8	0.018	90.9	96.4	0.129
N1	88.8	96.2	< 0.001	74.9	87.2	< 0.001	86.7	94.8	< 0.001	86.8	93.7	0.001
N2	89.7	94.7	0.144	70.6	77.5	0.160	85	85.4	0.655	78.3	85.8	0.07
N3	88.2	97.3	0.136	55.2	66.2	0.268	61	76.6	0.173	59.5	74.9	0.073
Overall												
I	-	98.7	-	-	97.6	-	-	100	-	-	98.9	-
II	100	95.6	0.749	100	88.5	0.612	100	97.9	0.837	100	94.6	0.739
III	90.8	96.6	0.002	77.7	86.1	0.003	90.3	91.9	0.137	87.1	92.4	0.024
IV	87.9	94.1	0.021	69.9	71.9	0.539	80.9	80.8	0.494	80.8	80.5	0.89

Abbreviations: PTI = primary tumor inflammation; LRFS = local relapse-free survival; DFS = disease-free survival; OS = overall survival; DMFS = distant metastases-free survival.

a *P* values were calculated using the log-rank test.

-No patients with stage I disease had PTI.

## DISCUSSION

To the best of our knowledge, this is the first large-scale study to investigate the prognostic value of PTI in NPC. In the present study, we observed a relatively high incidence (22.0%) of PTI, especially in patients with advanced *T* stage. The results of this study revealed that PTI was an independent prognostic factor with regard to 4-year LRFS, DFS and DMFS for patients with nonmetastatic NPC in the era of IMRT. However, no significant difference was found in 4-year OS for these two groups.

Due to the anatomical specificity and invasiveness, inflammation is common in the sphenoid sinus and nasal cavity in advanced T stage NPC and often combines with necrosis and bacterial infections, which results in local hypoxia and radioresistance. This was also observed in head and neck cancers [13–16]. Moreover, numerous previous studies showed that hypoxia is also an adverse prognostic factor in many malignant cancers like lung, breast, uterine cervix, rectum, brain, soft tissue and renal cell [17–23]. This mechanism may also explain the unfavorable prognostic value of cervical nodal necrosis in NPC [11].

In our cohort, patients with PTI tended to be older than patients without PTI, indicating that age may adversely contribute to primary tumor inflammation. One reasonable explanation is that older patients are likely to be in worse physical condition. PTI patients were also at a more advanced clinical stage on average, and tumor volume was generally larger. A greater number of PTI patients were thus undergoing chemotherapy. The significant prognostic difference showed by univariate analysis between these two groups should originate from unbalanced TNM staging and other prognostic factors. Multivariate analysis revealed PTI was an prognostic factor for LRFS, DFS and DMFS but not for OS, which indicated that the follow-up time was insufficient, and should be longer in any similar studies in future.

Subgroup analysis revealed a difference in the prognosis of PTI patients with T3 or N1 disease. Due to the extremely low incidence of PTI, no clinical prognostic value in T1-2 or N0 patients was observed. Moreover, patients with T4 or N2-3 are at a higher risk of distant metastases, and the prognostic impact of PTI may be masked by other important prognostic factors. The survival outcomes of PTI patients with T3 disease were similar to those with T4 classification. Hence, we proposed that T3 stage should be divided intoT3a and T3b stage based on PTI. More intensive chemotherapy regimen may be needed for patients with PTI compared with patients without PTI.

Another reason for unfavorable prognosis of PTI patients may be due to the difficulty in determining an accurate GTV using IMRT. Inflammation in the sphenoid sinus and nasal cavity often mixed with tumor and made it difficult to establish the tumor margin. Hence, the tumor target would easily be left out. Therefore, a thorough pretreatment assessment of PTI patients undergoing IMRT should be performed, and accurate delineation of GTV should be warranted to reduce local recurrence.

The findings of our current study suggest that clinicians should pay particular attention to NPC patients with PTI in order to accurately delineate the tumor target. Induction chemotherapy could be applied to shrink the tumor bulk and minimize inflammation to assist identification of the tumor margin. Additionally, a better dose coverage and reduced toxicity from radiotherapy could be achieved after induction chemotherapy. Additionally, proton and heavy particle therapy could result in better prognosis [24].

The main limitation of our study was that the judgement of PTI was only based on MRI. However, this could not be avoided because pathological results from surgical resection are not available due to the special location of NPC. The retrospective nature and short follow-up time are also limitations that should be addressed in future studies, and additional clinical experiments are needed to establish the prognostic value of PTI.

## CONCLUSIONS

In summary, this study confirmed that PTI was an independent prognostic factor for LRFS, DFS and DMFS for patients with NPC in the era of IMRT. It is advised that T3 stage should be divided into T3a and T3b stage based on the presence of PTI, and different intense treatment protocols should be considered for patients with PTI. Further prospective clinical study should be warranted to confirm the results of this current study.

## MATERIALS AND METHODS

### Patients

We retrospectively analyzed 1811 patients with newly diagnosed NPC that showed no evidence of distant metastasis, who were treated between November 2009 and February 2012 at Sun Yat-Sen University Cancer Center. Pre-treatment MR images of the nasopharynx and cervical region were thoroughly analyzed. Of the entire cohort, 103 (5.7%) patients without MRI results were excluded, which left 1708 (94.3%) patients for further investigation. This study was approved by the Research Ethics Committee of Sun Yat-Sen University Cancer Center, and informed consent was obtained from all patients.

### Clinical staging

The routine staging process included a complete history and clinical examination of the head and neck region, direct fibre-optic nasopharyngoscopy, MRI scans of the skull base, neck and chest radiography, a whole-body bone scan, and abdominal sonography. Positron emission tomography (PET)-CT scans were also performed if clinical indicated. All patients received a dental evaluation before radiotherapy.

All patients were restaged according to the 7th edition of the International Union against Cancer/American Joint Committee on Cancer (UICC/AJCC) system [25]. All MRI materials and clinical records were reviewed to minimize heterogeneity in restaging. Two radiologists employed at our hospital separately evaluated all of the scans and disagreements were resolved by consensus.

### Diagnostic criteria for PTI

**Figure 2:**
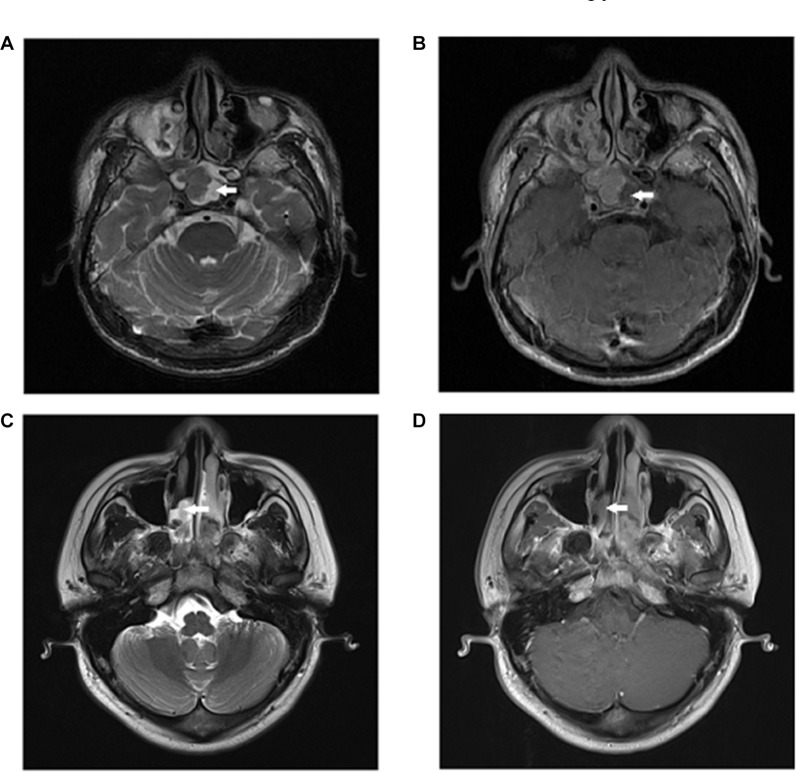
Primary tumor inflammation in two NPC patients (**A**) Axial T2-weighted and (**B**) contrast-enhanced T1-weighted MR images of a 41-year-old man show primary tumor inflammation in the sphenoid sinus; (**C**) Axial T2-weighted and (**D**) contrast-enhanced T1-weighted MR images of a 29-year-old man show primary tumor inflammation in the nasal cavity.

All patients underwent MRI scans using a 3 Tesla system (Trio Tim; Siemens, Erlangen Germany). Only patients with inflammation surrounding the tumor were included in this study, and patients with sinusitis not in the immediate vicinity of the tumor were excluded. Diagnostic criteria for primary tumor inflammation in MRI include an area of high signal intensity on T2-weighted images, and an area of low signal intensity on contrast material-enhanced T1-weighted images, which is similar to the diagnostic criteria for lymph node necrosis (Figure 2) [26, 27].

### Clinical treatment

#### Radiotherapy

All patients received intensity-modulated radiation therapy (IMRT) at Sun Yat-Sen University Cancer Center. Immobilization was carried out using a custom-made head-to-neck-thermoplastic cast with the patient's neck resting on a support. A high-resolution planning computed tomography scan with contrast was taken from the vertex to 2 cm below the sternoclavicular joint at a slice thickness of 3 mm. Target volumes were delineated slice-by-slice on treatment planning CT scans using an individualized delineation protocol that complies with International Commission on Radiation Units and Measurements report numbers 50 and 62. The prescribed doses were 66–72 Gy at 2.12–2.43 Gy/fraction to the planning target volume (PTV) of the primary gross tumour volume (GTVnx), 64–70 Gy to the PTV of the GTV of the involved lymph nodes (GTVnd), 60–63 Gy to the PTV of the high-risk clinical target volume (CTV1), and 54–56 Gy to the PTV of the low-risk clinical target volume (CTV2). All targets were treated simultaneously using the simultaneous integrated boost technique.

### Chemotherapy

According to our institutional guidelines, prior to commencing treatment we recommended radiotherapy alone for stage I disease, concurrent chemoradiotherapy (CCRT) for stage II disease, and CCRT +/− neoadjuvant/adjuvant chemotherapy for stage III to IVA-B disease. Neoadjuvant or adjuvant chemotherapy consisted of cisplatin with 5-fluorouracil, cisplatin with taxoids or cisplatin with 5-fluorouracil and toxoids, every three weeks for two or three cycles. Concurrent chemotherapy consisted of cisplatin given weekly or on weeks 1, 4 and 7 of radiotherapy.

### Follow-up and statistical analysis

Patients were followed-up from the first day of therapy to the day of last examination or death, and were examined at least every three months during the first two years, with follow-up examinations every six months thereafter until death. The end points (time to the first defining event) included LRFS, DFS, OS, and DMFS. LRFS was chosen as the first endpoint in this study.

Chi-square or Fisher exact tests were used to compare the categorical characteristics and treatment failure patterns between PTI and non-PTI groups, and subgroups were analyzed according to T classification, N classification, and overall stages. Life-table estimation was performed using the Kaplan-Meier method and differences were compared using the log-rank test. The multivariate Cox proportional hazards model was used to estimate the hazard ratio (HR) and to calculate 95% confidence intervals (CI). Variables in the model included age, gender, pathology, T classification, N classification, chemotherapy, and PTI. All statistical tests were two-sided, and *P* < 0.05 was considered statistically significant. The STATA statistical package (STATA 12; StataCorp LP, College Station, TX, USA) was used for all analyses.
